# Cinnamic Acid: A Shield Against High-Fat-Diet-Induced Liver Injury—Exploring Nrf2’s Protective Mechanisms

**DOI:** 10.3390/ijms26167940

**Published:** 2025-08-17

**Authors:** Asmahan Taher Alahdal, Laila Naif Al-Harbi, Ghedeir M. Alshammari, Ali Saleh, Mohammed Abdo Yahya

**Affiliations:** Department of Food Science and Nutrition, College of Food and Agricultural Sciences, King Saud University, Riyadh 11451, Saudi Arabia; 443203940@student.ksu.edu.sa (A.T.A.); aghedeir@ksu.edu.sa (G.M.A.); 442106909@student.ksu.edu.sa (A.S.); mabdo@ksu.edu.sa (M.A.Y.)

**Keywords:** cinnamic acid, Nrf2, hepatoprotection, high-fat diet, oxidative stress

## Abstract

This study investigated the hepatoprotective effects of cinnamic acid (CA) against liver injury and fat accumulation induced by a high-fat diet (HFD), focusing on the role of the nuclear factor erythroid 2-related factor 2 (Nrf2) signaling. Male Wistar rats were divided into six groups: a control group receiving carboxymethylcellulose; a CA control group (40 mg/kg); an HFD group; two HFD groups treated with CA (20 mg/kg or 40 mg/kg); and a HFD group co-treated with CA (40 mg/kg) and brusatol (2 mg/kg, i.p.), a selective Nrf2 inhibitor. CA was administered orally, and brusatol intraperitoneally, both twice per week for twelve weeks. CA had no effect on serum glucose or insulin but improved serum and hepatic profiles in HFD rats. It also attenuated liver vacuolization and normalized serum levels of ALT, AST, and γ-GT. CA also reduced hepatic apoptosis by increasing Bcl2 and reducing Bax and caspase-3 levels. CA mitigated oxidative stress by reducing MDA and enhancing SOD and GSH levels. It suppressed inflammatory mediators, including TNF-α, IL-6, and NF-κB. CA also downregulated SREBP1, FAS, ACC-1, and Keap1 while increasing mRNA and nuclear translocation of Nrf2. All these effects were dose-dependent. Similar molecular effects of CA were also seen in control rats while CA protection in HFD rats was abolished with brusatol indicating Nrf2-dependency. Such findings highlight CA as a promising nutraceutical candidate for preventing HFD-induced liver injury. Further studies are warranted to explore its clinical applicability in metabolic liver diseases.

## 1. Introduction

Obesity represents a major global health challenge, significantly impacting public health and socioeconomic stability. In Saudi Arabia, approximately 34% of the population is obese, contributing to a surge in related conditions such as type 2 diabetes, cardiovascular disease, and metabolic syndrome [1,2]. Among these, non-alcoholic fatty liver disease (NAFLD) is particularly concerning, marked by liver fat accumulation in the absence of alcohol intake [2]. NAFLD ranges from simple steatosis to non-alcoholic steatohepatitis (NASH), with potential progression to hepatocellular carcinoma [3]. Notably, over 50% of obese individuals develop NAFLD, and 80% of these may advance to NASH [3,4].

The pathogenesis of non-alcoholic fatty liver disease (NAFLD) within the context of obesity is complex and multifactorial, encompassing both peripheral and hepatic mechanisms. NAFLD initiation and progression involve various cellular processes, including the activation of Kupffer cells, increased inflammatory cytokine production, heightened reactive oxygen species (ROS) generation, antioxidant depletion, and mitochondrial dysfunction [5]. Key alterations in transcription factors, such as carbohydrate response element-binding protein (ChREBP) and sterol regulatory element-binding protein 1 (SREBP-1), also play vital roles in NAFLD pathogenesis [6,7]. Central to this process is insulin resistance (IR), establishing a dual-hit model of liver injury and steatosis [2,8]. According to this, the first hit is induced by hepatic accumulation, which can be exaggerated by increasing the influx of free fatty acids from insulin-resistant tissue to promote a second hit induced by oxidative stress and inflammation [8].

The Kelch-like ECH-associated protein 1/nuclear factor erythroid 2-related factor 2 (Keap1/Nrf2) signaling pathway is pivotal in cellular responses to oxidative stress, serving as a cornerstone for cell survival [9]. Nrf2, a crucial transcription factor, orchestrates the expression of glutathione (GSH) and essential phase two antioxidant enzymes such as superoxide dismutase (SOD), heme oxygenase-1 (HO-1), and catalase, by binding to antioxidant response elements (AREs) within the nucleus [9,10]. Under normal physiological conditions, Nrf2 is subjected to ubiquitination-mediated degradation by Keap1. However, oxidative stress induces modifications in Keap1, leading to Nrf2 stabilization, nuclear translocation, and activation [9]. Recent studies illuminate Nrf2′s vital key role in NAFLD pathogenesis by preserving hepatic redox balance, inhibiting inflammation, and modulating metabolic processes such as fatty acid oxidation, lipogenesis, and gluconeogenesis [11,12,13]. Indeed, Nrf2 not only boosts antioxidants but suppresses lipogenesis by inhibiting major lipogenic genes such as the peroxisome proliferator-activated receptor alpha (PPARα), fatty acid synthase (FAS), and acetyl-CoA carboxylase (ACC) and stimulates mitochondrial biogenesis and FA oxidation through stimulating the activation and the expression of the peroxisome proliferator-activated receptor alpha (PPARα) [11,12,13,14,15]. Notably, Nrf2 signaling is largely impaired in the liver of rodents with NAFLD and was associated with increased generation of ROS and inflammatory cytokines, reduced levels of antioxidant enzymes, impaired hepatic FA oxidation, enhanced lipogenesis, and increased lipid accumulation and steatosis [16]. Also, Nrf2 deficiency impairs fatty acid oxidation and exacerbates hepatic steatosis in murine models [14,17,18,19]. Activation of Nrf2 alleviated NAFLD symptoms and liver damage, suggesting that compounds modulating the Keap1/Nrf2 signaling pathway may offer valuable therapeutic avenues for NAFLD prevention and management [15,19,20].

Current management strategies for NAFLD predominantly emphasize lifestyle modifications and insulin-sensitizing medications [21]. Emerging evidence suggests that plant-derived polyphenols could provide substantial therapeutic benefits due to their antioxidant and anti-inflammatory properties and their ability to modulate various signaling pathways [22,23]. Cinnamic acid (CA), a naturally occurring phenolic compound found in foods such as cinnamon, spinach, tea, and citrus fruits, has attracted considerable scientific interest for its treatment of various human diseases, including liver disorders, due to its unique pharmacological properties [24,25]. Research indicates that CA effectively mitigates cardiac, neural, and renal damage in several animal models by suppressing ROS and inflammatory cytokine production while enhancing antioxidant defenses [26,27]. Also, CA’s showed potent potential to improve insulin sensitivity in obese rats, reinforcing its therapeutic promise for metabolic disorders [28].

Of interest, CA attenuated NAFLD by downregulating fat synthesis genes such as fatty acid synthase (FAS) and Acetyl CoA Carboxylase (ACC-1), while promoting fatty acid oxidation [29]. Despite these promising findings, the precise mechanisms of CA’s action on NAFLD remain to be fully elucidated. Further exploration of CA’s mechanisms is warranted to fully understand its therapeutic potential in NAFLD. Notably, CA acts as a potent activator of Nrf2, crucial for regulating cellular antioxidant responses [30,31]. It also protected against acute hepatitis by modulating Nrf2 and NF-κB signaling pathways [32].

Whether cinnamic acid (CA) can alleviate hepatic steatosis and injury in NAFLD by modulating Nrf2 remains unclear. Therefore, this study aimed to investigate the antioxidant, anti-inflammatory, and anti-lipogenic effects of CA in a rat model of HFD-induced NAFLD. We specifically tested the hypothesis that CA mediates its protective effects through Nrf2 activation, leading to the regulation of key downstream targets, including antioxidant enzymes and lipogenic transcription factors SREBP1, PPARα, FAS, and ACC.

## 2. Results

### 2.1. CA Fails to Mitigate Fasting Glucose Levels During the IPGTT in HFD Rats

**Figure 1:**
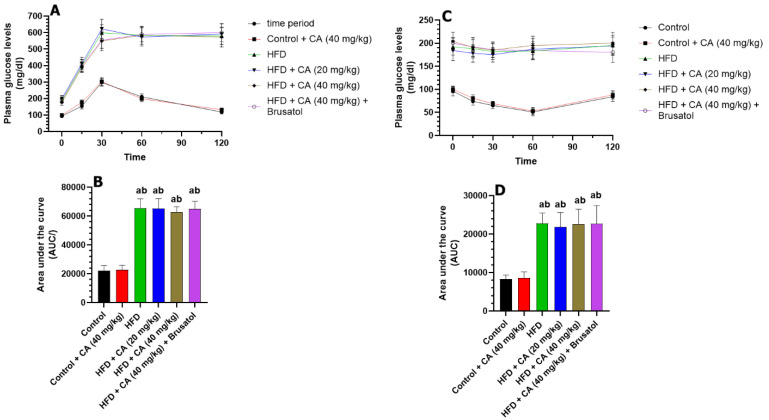
(**A**,**B**) Fasting plasma glucose during the intraperitoneal glucose tolerance test (IPGTT) and their corresponding area under the curve, respectively, in all experimental groups. (**C**,**D**): Fasting plasma glucose during the intraperitoneal insulin tolerance test (IPITT) and their corresponding area under the curve, respectively, in all experimental groups. (**A**): significantly different as compared to control rats; (**B**): significantly different as compared with control + CA (40 mg). Normality was tested using the Shapiro–Wilk test. In (**A**,**C**), data were analyzed by 2-way ANOVA with multiple measures. In (**C**), data were analyzed using 2-way ANOVA followed by Tukey’s *t*-test as post hoc. All data are presented as means + SD for n = 8 rats/groups. a: significantly different vs. control; b: significantly different vs. control + CA (20 mg/kg).

The changes in plasma glucose levels over the 120 min post the IPGTT and their corresponding area under the curve (AUC) are shown in Figure 1A,B. Treating control rats with CA (40 mg/kg) resulted in non-significant changes in fasting plasma glucose levels at 0, 15, 30, 60, and 120 min, showing increases of +6.5%, +14.9%, +1.7%, +1.1%, and +8.8%, respectively (all *p* > 0.05). In contrast, the HFD group exhibited significant increases in measured plasma glucose levels with changes of +100% at 0 min, +159.1% at 15 min, +100.3% at 30 min, +113.4% at 60 min, and +109.2% at 120 min (all *p* < 0.001) as compared to control rats. Both HFD + CA (20 mg/kg) and HFD + CA (40 mg/kg) showed negligible, non-significant changes at all time points. The addition of brusatol (an Nrf2 inhibitor) to HFD + CA (40 mg/kg) yielded non-significant effects as compared to HFD + CA (20 mg/kg), HFD + CA (40 mg/kg), and HFD rats. Additionally, AUC analysis confirmed these results, revealing a slight, non-significant increase of 1.43% for control + CA (40 mg/kg) versus control (*p* > 0.05). HFD rats showed a significant increment in the AUC increase of 194.59% (*p* < 0.001) as compared to control rats. Finally, HFD + CA (40 mg/kg) + brusatol exhibited a modest, non-significant AUC increase of 3.02% compared to HFD + CA (40 mg/kg) (*p* > 0.05), which was also not significant when compared to HFD + CA (20 mg/kg) or to HFD rats.

The changes in plasma glucose levels over the 120 min post the IPITT and their corresponding area under the curve (AUC) are shown in Figure 1C,D. Treatment with CA (40 mg/kg) in control rats resulted in non-significant changes in plasma glucose levels compared to the control group: +4.2% at 0 min, +9.5% at 15 min, +6.2% at 30 min, +3.9% at 60 min, and +4.8% at 120 min (all *p* > 0.05). The corresponding AUC analysis also showed a non-significant increase of +1.03% (*p* > 0.05) when control + CA (40 mg/kg) was compared to control rats. In contrast, the HFD group exhibited significant increases in plasma glucose levels relative to the control group: +100% at 0 min, +154.1% at 15 min, +180% at 30 min, +258.8% at 60 min, and +132.1% at 120 min (all *p* < 0.001). This produced a significant increase in the UAC (+202.3%; *p* < 0.001) as compared to the control group. When comparing HFD + CA (20 mg/kg) to the HFD group, changes were also not significant with +4.2% at 0 min, −5.3% at 15 min, −6.5% at 30 min, −7.0% at 60 min, and −4.8% at 120 min (all *p* > 0.05). Similarly, HFD + CA (40 mg/kg) showed non-significant changes in plasma glucose levels at all measure intervals: +6.2% at 0 min, −3.9% at 15 min, −6.1% at 30 min, −8.2% at 60 min, and −7.7% at 120 min compared to the HFD group (all *p* > 0.05). The resulting UAC for CA (20 mg/kg) and HFD + CA (40 mg/kg) showed non-significant changes of −1.35% (*p* > 0.05) and −4.54% (*p* > 0.05), respectively, as compared to HFD. In the same manner, the levels of plasma glucose measured at all time intervals and their areas under the curve in HFD + CA (40 mg/kg) + brusatol-treated rats were also not significantly different as compared to HFD, HFD + CA (20 mg/kg) or HFD + CA (40 mg/kg) groups.

### 2.2. CA Does Not Change Adiposity Markers in HFD Rats

When comparing the control group with control + CA (20 mg/kg), CA did not cause any significant changes in initial body weight (−1.2%), final body weight (+0.68%), weight gain (+2.99%), cumulative food intake (+2.93%), calorie intake (+2.25%), BMI (−4.41%), total fat weight (−2.53%), or adiposity index (+5.56%) (all *p* > 0.05) (Table 1). In contrast, the HFD group showed significant increases in their final body weight (+33.6%, *p* < 0.001), weight gain (+53.5%, *p* < 0.001), cumulative food intake (+56.8%, *p* < 0.001), calorie intake (+113.5%, *p* < 0.001), BMI (+23.5%, *p* < 0.001), total fat weight (+80.6%, *p* < 0.001), and adiposity index (+33.3%, *p* < 0.001) as compared to control rats (Table 1). However, and as compared to HFD-fed rats, HFD + CA (20 mg/kg) or HFD + CA (40 mg/kg) exhibited no significant changes in rats’ final body weight (−2.8% and −2.5%) and weight gain (−4.6% and −9.4%), cumulative food intake (+3.7% and +1.4%), calorie intake (+3.7% and +1.3%), BMI (+2.4% and −1.2%), total fat weight (+4.9% and +2.1%), or adiposity index (+8.3% and +2.8) (all *p* > 0.5) (Table 1). In the same manner, HFD + CA (40 mg/kg) + brusatol-treated rats showed no significant alterations in the final body weight (−4.9%), weight gain increased (+16.0%), cumulative food intake (+0.5%), calorie intake (+0.1%), BMI (+2.4%), total fat weight (−2.5%), and adiposity index (+1.4%) (all *p* > 0.05), as compared to HFD + CA (40 mg/kg) (Table 1). The levels of all these measurements were also not significantly different when HFD + CA (40 mg/kg) + brusatol-treated rats were compared to HFD rats.

### 2.3. CA Fails to Alter Fasting Levels of Glucose, Insulin, and HOMA-IR

Fasting plasma glucose and insulin levels, as well as the values of HOMA IR were not significantly varied between the control + CA (40 mg/kg) and control groups showing percentages of changes of +1.76%, 10.26%, and −7.69%, respectively (all *p* > 0.05) (Table 2). In stark contrast, the HFD group exhibited significant increases in the levels of these measured parameters as compared to control + CA (40 mg/kg), with fasting plasma glucose rising by +105.50%, fasting plasma insulin by +117.14%, and HOMA-IR by +339.29% (all *p* < 0.01), as compared to control rats highlighting a clear state of IR (Table 2). The addition of CA (20 mg/kg) or (40 mg/kg) to the HFD group resulted in non-significant changes (all *p* > 0.05) in fasting plasma glucose (−1.88% and +2.03%), fasting plasma insulin (+ 3.95% and −2.63%), and HOMA-IR (+4.60% and 1.68%), respectively, as compared to HFD rats (Table 2). Also, the co-administration of brusatol to the HFD + CA (40 mg/kg) group resulted in a non-significant decrease in fasting glucose (−5.81%), as well as non-significant increases in fasting insulin (+1.35%) and HOMA-IR (+1.68%) (all *p* > 0.05), as compared to HFD + CA (40 mg/kg) (Table 2). Also, the fasting levels of plasma glucose, insulin, and HOMA-IR were not significantly different between the HFD + CA (40 mg/kg) + brusatol as compared to HFD rats.

### 2.4. CA Lowers Serum and Hepatic Lipids in Control and HFD Rats

Control + CA (40 mg/kg) group showed a significant reduction in the levels of serum levels of TGs (−26.54%), CHOL (−27.76%), and LDL-c (−26.89%), as well as in hepatic levels of TGs (−26.79%) and CHOL (−21.62%) (all *p* < 0.05) as compared to control rats, indicating a beneficial effect of CA on lipid profiles under basal conditions (Table 2). In contrast, the HFD group exhibited significant increases in serum levels of TGs (+205.34%), CHOL (+199.36%), and LDL-c (+205.34%), as well as in hepatic TGs (+237.80%) and hepatic CHOL (+201.72%) (all *p* < 0.001) as compared to control rats (Table 2). When compared to HFD rats, the addition of CA (20 mg/kg) and CA (40 mg/kg) to the HFD resulted in significant decreases in serum TGs (−34.19% and 53.37%), serum CHOL (−22.98% and −51.41%), serum LDL-c (−29.36% and −52.39%), hepatic TGs (−31.88% and −55.51%), and hepatic CHOL (−30.29% and −57.14%) (all *p* < 0.01), respectively. Opposing this, HFD + CA (40 mg/kg) + brusatol group of rats showed significant (all *p* < 0.01) in serum levels of TGs (+121.14%), CHOL (+99.20%), and LDL-c (+100.00%), alongside significant increases (all *p* < 0.01) in hepatic TGs (+109.84%) and hepatic CHOL (+146.67%) as compared to HFD + CA (40 mg/kg) as compared to HFD + CA (40 mg/kg) (Table 2). However, no significant changes in the serum or hepatic levels of all these lipids were seen when comparing HFD to HFD + CA (40 mg/kg) + brusatol.

### 2.5. CA Attenuates the Increments in Serum Function Enzymes and Apoptosis Markers

No significant alterations in the serum levels of ALT, AST, and γ-GT (−11.61%) in hepatic levels of Bax and cytochrome-c were observed between the control and control + CA (40 mg/kg)-treated groups (all *p* > 0.05) (Table 3). However, levels of BCL2 were significantly higher in the liver homogenates of control + CA (40 mg/kg) (+41.81%) as compared to control rats (*p* < 0.001) (Table 3). HFD-fed rats demonstrated pronounced hepatic injury, with significant elevations in serum ALT (+192.41%), AST (+152.81%), and γ-GT (+207.59%) relative to controls (all *p* < 0.001) (Table 3). These changes coincided with marked pro-apoptotic shifts, as evidenced by substantial reductions in hepatic Bcl2 (−54.71%) and dramatic increases in Bax (+395.89%) and caspase-3 (+324.53%) (all *p* < 0.001). Treatment with HFD + CA (20 mg/kg) markedly reversed these alterations. Serum ALT, AST, and γ-GT levels were reduced by −33.03%, −24.76%, and −39.47%, respectively, versus HFD rates (all *p* < 0.01) (Table 3). Apoptotic stress was also alleviated, with Bax (−27.65%) and caspase-3 (−44.89%) levels significantly reduced, and Bcl-2 increasing by +49.39% (all *p* > 0.001) (Table 3). Nonetheless, HFD + CA (40 mg/kg) treatment exerted more potent effects, nearly normalizing hepatic function, a robust restoration in BCl2 (+102.02%), and substantial declines in Bax (−70.15%) and caspase-3 (−72.00%) (Table 3).

### 2.6. CA Alleviates Oxidative Stress and Inflammation in the Livers of HFD Rats and Stimulates Antioxidant Levels in the Livers of Both the Control and HFD Rats

**Figure 2:**
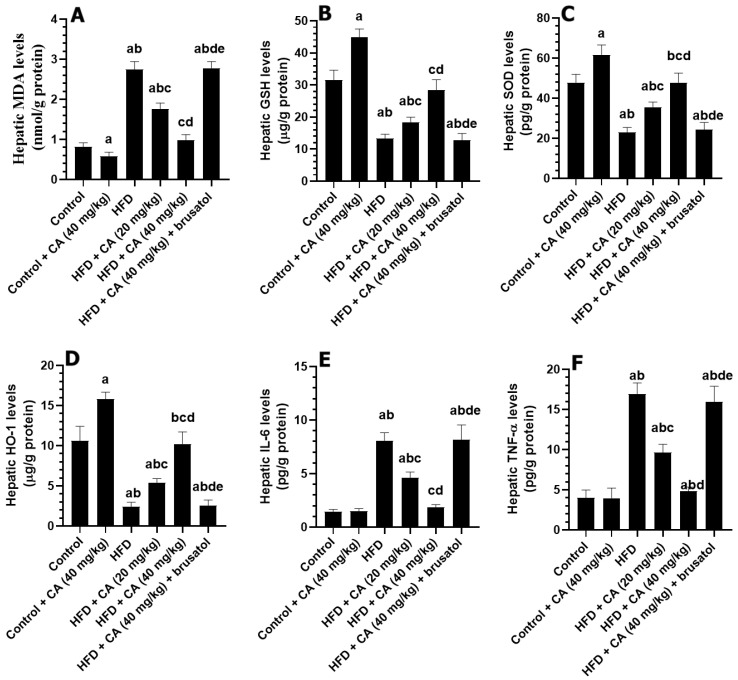
Levels of some oxidative stress (**A**–**C**) and inflammation (**D**–**F**) in the livers of all experimental groups. Normality was tested using the Shapiro–Wilk test. Data were analyzed by one-way followed by Tukey’s *t*-test as post hoc. Data are given as the means ± SD of 8 rats/group. Data are given as the means ± SD of 8 rats/group. The level of significance was shown as *p* < 0.05. a: significantly different vs. control, b: significantly different vs. control + CA (20 mg/kg), c: significantly different vs. HFD, d: significantly different vs. HFD + CA (20 mg/kg), and e: significantly different vs. HFD + CA (40 mg/kg).

As compared to control rats, the livers of the control + CA (40 mg/kg) demonstrated a significant reduction in levels of MDA (−30.77%) with a parallel increase in the levels of SOD (+26.09%), GSH (+25.71%), and HO-1 (+83.33%) (all *p* < 0.01) (Figure 2A–F). Conversely, hepatic IL-6 and TNF-α were non-significantly decreased by −5.88% and 6.75%, respectively (both *p* > 0.05), in the livers of these rats as compared to control rats. In contrast, and as compared to control rats, the livers of the HFD group exhibited a significant elevation in MDA by +381.48%, IL-6 by +385.51%, and TNF by +190.57%, while GSH and SOD decreased by −65.00% and −61.55%, respectively (all *p* < 0.001) (Figure 2A–F). The HFD + CA (20 mg/kg) treatment resulted in a reduction in the hepatic levels of MDA (−33.65%) and IL-6 (−48.81%) with a concomitant increase in the hepatic levels of GSH (+25.97%), SOD (+48.46%), and HO-1 (+100%) (all *p* < 0.001) as compared to HFD (Figure 2A–F). The higher dose, HFD + CA (40 mg/kg), led to further reductions in the hepatic levels of MDA (−68.08%) and IL-6 (−83.01%) with more profound significant increases in the hepatic levels of GSH (+62.34%), SOD (+118.93%), and HO-1 (+222.22%) (all *p* < 0.001) (Figure 2A–F). However, the liver of the HFD + CA (40 mg/kg) + brusatol rats showed an increase in the levels of MDA by +225.30% (*p* < 0.001) and TNF by +280.49% (*p* < 0.001), alongside significant decreases in the levels of GSH (−33.20%), SOD (−56.33%), and HO-1 (−71.69%) (all *p* < 0.001) (Figure 2A–F). Interestingly, no significant changes in the levels of all these parameters were observed between the HFD and HFD + CA (40 mg/kg) + brusatol groups.

### 2.7. CA Enhances the Hepatic Nuclear Translocation of Nrf2 by Suppressing the Expression of Keap-1 Under Basal and HFD Conditions

**Figure 3:**
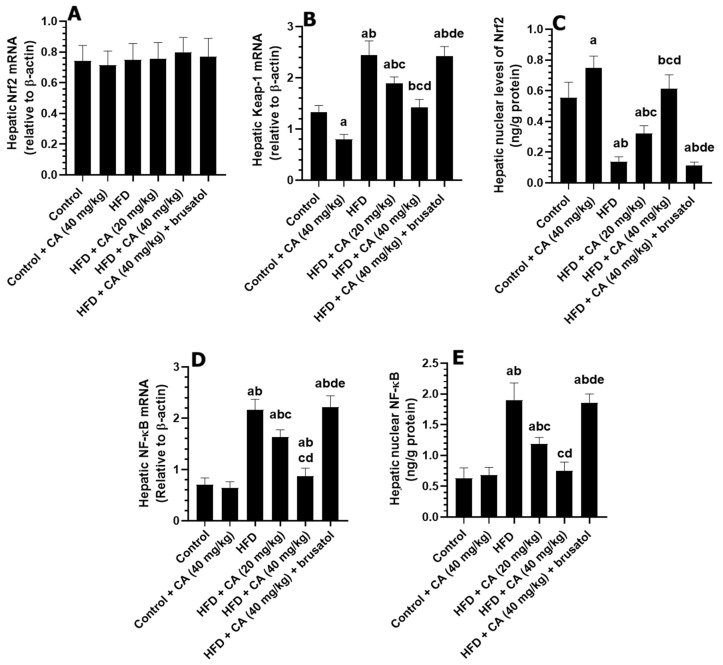
The effect of all treatments on hepatic mRNA of Nrf2 (**A**), Keap1 (**B**), and NF-κB (**D**), as well as nuclear protein levels of Nrf2 (**C**) and NF-κB (**E**). Normality was tested using the Shapiro–Wilk test. Data were analyzed by one-way followed by Tukey’s *t*-test as post hoc. Data are given as the means ± SD of 8 rats/group. Data are given as the means ± SD of 8 rats/group. The level of significance was shown as *p* < 0.05. a: significantly different vs. control, b: significantly different vs. control + CA (20 mg/kg), c: significantly different vs. HFD, d: significantly different vs. HFD + CA (20 mg/kg), and e: significantly different vs. HFD + CA (40 mg/kg).

The mRNA levels of Nrf2 were not significantly different among all groups of rats. The livers of the control + CA (40 mg/kg) group showed a slight non-significant reduction in mRNA levels of NF-kB (−1.49%, *p* > 0.05) and in the nuclear levels of NF-kB (+12.46%, *p* > 0.05), as compared to control rats but showed a significant reduction in mRNA levels of Keap1 (−39.55%, *p* < 0.01) and an increase in nuclear levels of Nrf2 (+29.41%, *p* < 0.01) (Figure 3A–E). When comparing the livers of the HFD group to those of the control group, significant increases were observed in mRNA levels of NF-kB (+209.85%, *p* < 0.001), mRNA levels of Keap1 (+80.60%, *p* < 0.001), and nuclear levels of NF-kB (+190.48%, *p* < 0.001), which were associated with a significant reduction in nuclear Nrf2 levels (−66.67%, *p* < 0.001). The analysis of the HFD + CA (20 mg/kg) group against the HFD group revealed notable reductions in mRNA levels of NF-kB (−22.10%, *p* < 0.05) and Keap1 (−22.36%, *p* < 0.05), along with a significant decrease in nuclear levels of NF-kB (−34.89%, *p* < 0.05) and a notable increase in nuclear Nrf2 levels (+47.06%, *p* < 0.01). In the comparison of HFD + CA (40 mg/kg) to the HFD group, marked reductions were observed in mRNA levels of NF-kB (−59.62%, *p* < 0.001), nuclear levels of NF-kB (−60.11%, *p* < 0.001), and Keap1 mRNA (−42.62%, *p* < 0.01), alongside a substantial and significant increase in nuclear Nrf2 levels (+252.94%, *p* < 0.001) (Figure 3A–E). Conversely, when comparing the HFD + CA (40 mg/kg) group to the HFD + CA (40 mg/kg) + brusatol group, significant increases were observed in mRNA levels of NF-kB (+167.86%, *p* < 0.001), nuclear levels of NF-kB (+150.68%, *p* < 0.001), and mRNA levels of Keap1 (+83.57%, *p* < 0.001), while nuclear Nrf2 levels significantly decreased (−83.33%, *p* < 0.01) (Figure 3A–E).

### 2.8. CA Downregulates Hepatic Lipogenic Genes and Stimulates PPARα in Both the Control and HFD Rats

**Figure 4:**
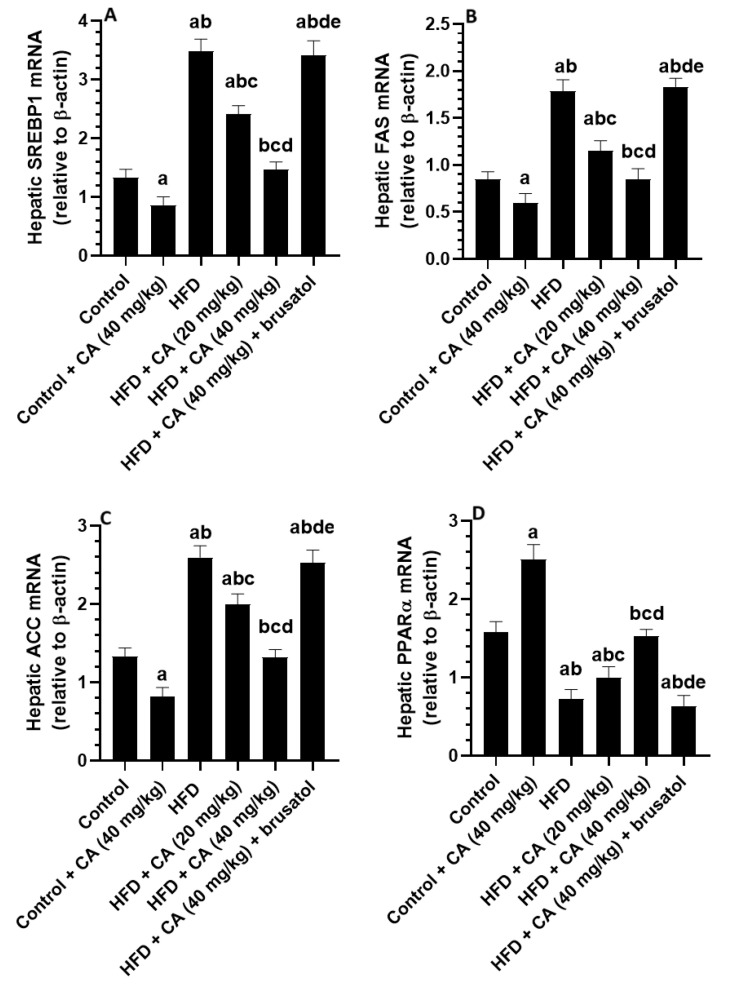
Expression levels of mRNA of some lipogenic genes (**A**–**D**) in the livers of all experimental groups. Normality was tested using the Shapiro–Wilk test. Data were analyzed by one-way followed by Tukey’s *t*-test as post hoc. Data are given as the means ± SD of 8 rats/group. Data are given as the means ± SD of 8 rats/group. The level of significance was shown as *p* < 0.05. a: significantly different vs. control, b: significantly different vs. control + CA (20 mg/kg), c: significantly different vs. HFD, d: significantly different vs. HFD + CA (20 mg/kg), and e: significantly different vs. HFD + CA (40 mg/kg).

Compared to control rats, administration of CA (40 mg/kg) to control rats resulted in significant decreases in the hepatic mRNA levels of SREBP1 (−37.50%), FAS (−44.58%), and ACC (−40.44%), while mRNA PPARα levels were significantly increased (+64.29%) (all *p* < 0.001) (Figure 4A–D). Conversely, HFD feeding led to significant increases in the mRNA levels of SREBP1 (+152.94%), FAS (+109.64%), and ACC (+90.44%) compared to control rats, with a significant reduction in hepatic mRNA levels of PPARα (−56.52%, *p* < 0.001) (Figure 4A–D). The administration of CA (20 mg/kg) against HFD revealed a significant reduction in the hepatic mRNA levels of SREBP1 (−30.93%, *p* < 0.01), FAS (−28.74%, *p* < 0.05), and ACC (−21.96%, *p* < 0.05), alongside an increase in mRNA levels of PPARα (+41.79%, *p* < 0.01) as compared to HFD rats (Figure 4A–D). Treatment with CA (40 mg/kg) showed a further and more profound decrease in the mRNA levels of SREBP1 (−57.80%), FAS (−58.16%), and ACC (−48.34%) compared to the HFD group, while PPARα levels significantly increased (+118.78%) (all *p* < 0.001) (Figure 4A–D). Opposing to this, the combination of CA (40 mg/kg) with brusatol led to significant increases in mRNA levels of SREBP1 (+142.07%), FAS (+164.38%), and ACC (+87.31%) compared to the HFD + CA (40 mg/kg) group, while PPARα expression decreased (−55.84%) (all *p* < 0.001) (Figure 4A–D).

### 2.9. CA Prevents Hepatic Damage and Restores Normal Liver Histology in HFD Rats

**Figure 5:**
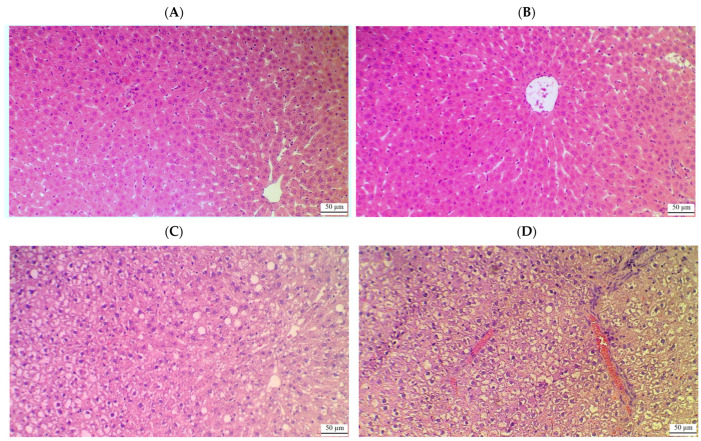
Histological images of the livers from control (**A**), control + CA (40 mg/kg) (**B**), and HFD rats (**C**,**D**). (**A**,**B**) show normal histological images with well-defined and normally appearing central veins, normally sized sinusoids, and intact hepatocytes radiating from the central vein with round intact nuclei. (**C**,**D**) show several abnormalities, including the presence of cytoplasmic vacuoles in the majority of the hepatocytes that were concomitant with an increased number of fat vacuoles, pyknotic nuclei, immune cell infiltration, and hemorrhage.

**Figure 6:**
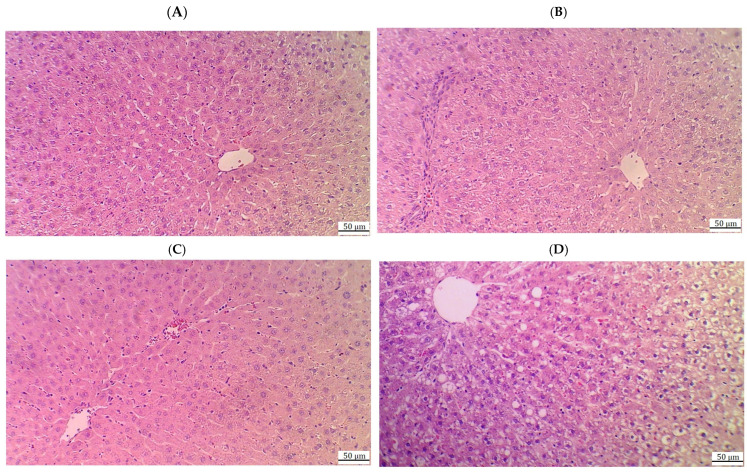
Histological images of the livers from the HFD + CA (20 mg/kg) (**A**,**B**), HFD + CA (40 mg/kg) (**C**), and HFD + CA (40 mg/kg) + brusatol rats (**D**). (**A**,**B**) show much improvement in the livers of these rats with an increased number of normal hepatocytes with intact nuclei and reductions in the cytoplasmic vacuolization and absence of fat vacuoles. However, some damage to the central vein with immune infiltration is still visible; (**C**) shows an almost normal liver structure with intact central veins, hepatocytes, and nuclei, as well as normally sized sinusoids and very few cytoplasmic vacuolizations. (**D**) demonstrates obvious damage in the central vein and the swelling of hepatocytes with increased numbers of fat vacuoles, where vacuolization was seen in the majority of cells. Pyknotic nuclei were also dominant in a large number of cells.

Control rats (Figure 5A) and those treated with 40 mg/kg of CA (Figure 5B) exhibited normal liver architecture, characterized by well-defined central veins, appropriately sized sinusoids, and intact hepatocytes radiating from the central vein, with round, intact nuclei. In contrast, rats subjected to a high-fat diet (HFD) (Figure 5C,D) displayed significant histological abnormalities, including widespread cytoplasmic vacuolization in hepatocytes, an increased number of fat vacuoles, pyknotic nuclei, immune cell infiltration, and evidence of hemorrhage. Further evaluation of the HFD groups treated with varying doses of CA revealed improvements in liver histology. Rats receiving 20 mg/kg of CA (Figure 6A,B) showed a marked increase in normal hepatocytes with intact nuclei, along with a reduction in cytoplasmic vacuolization and the absence of fat vacuoles. However, some damage to the central vein and residual immune infiltration were still present. The group treated with 40 mg/kg of CA (Figure 6C) exhibited nearly normal liver structure, with intact central veins and hepatocytes, normally sized sinusoids, and minimal cytoplasmic vacuolization. Conversely, the group receiving both 40 mg/kg of CA and brusatol (Figure 6D) demonstrated significant damage to the central vein and hepatocyte swelling, with a notable increase in fat vacuoles and prevalent pyknotic nuclei throughout the majority of cells.

## 3. Discussion

**Figure 7:**
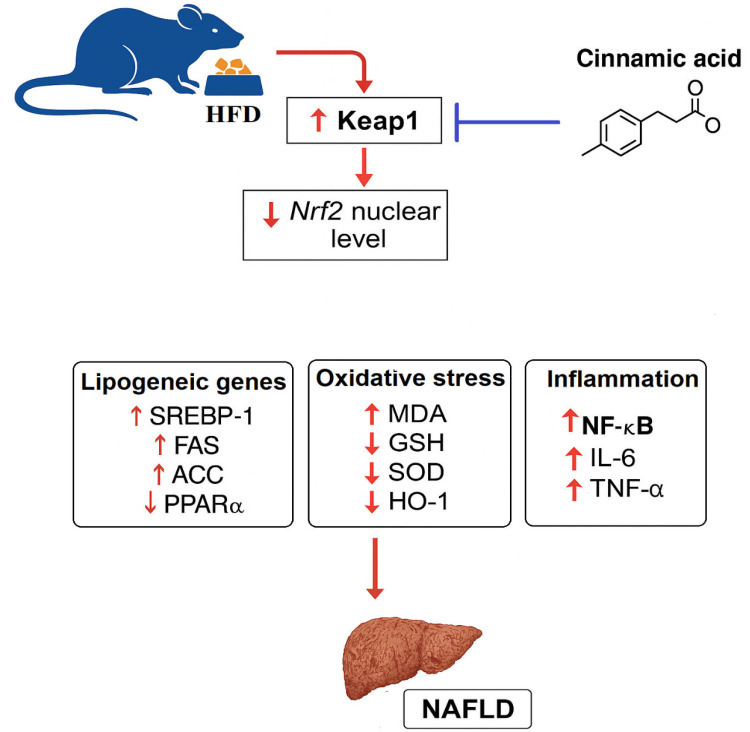
A graphical abstract shows the therapeutic effects of cinnamic acid against high-fat-diet-induced liver injury.

This study examines the therapeutic potential of CA as a pharmacological agent for treating hepatic steatosis and NAFLD in rats fed a HFD. Our findings indicate that CA significantly reduces hepatic lipogenesis, oxidative stress, and inflammation by increasing antioxidants such as GSH, SOD, and HO-1. Additionally, CA decreases pro-inflammatory cytokine production and inhibits key lipogenic transcription factor, SREBP1, along with its downstream enzymes, FAS and ACC-1, while enhancing PPARα activity. Importantly, these effects are dose-dependent and are not influenced by body weight, insulin sensitivity, or glucose metabolism. Mechanistically, CA’s benefits are mediated through Nrf2 activation, linked to reduced Keap1 expression, which suggests the potential for targeted liver health interventions (Figure 7).

The HFD animal model is pivotal for studying metabolic disturbances and NAFLD, as it closely mirrors the metabolic disturbances seen in human conditions [33,34]. This model enables the exploration of therapeutic interventions and elucidates the mechanisms driving hyperglycemia and IR in relation to NAFLD, thus serving as a cornerstone in metabolic disease research [35]. Chronic HFD consumption leads to excessive fatty acid influx into peripheral tissues such as the muscles and adipose tissue, impairing fatty acid oxidation while elevating oxidative stress and inflammation. Such disruption of insulin signaling results in the expansion of fat mass, obesity, and reduced insulin sensitivity, which directly contribute to IR and hyperglycemia [36]. In addition, the increments in the influx of adipose tissue-derived FFAs to the liver impair mitochondrial function and generate massive amounts of ROS and inflammatory cytokines, which in turn induce central hepatic IR. This further worsens hyperglycemia by increasing hepatic glucose production through stimulating gluconeogenesis and glycogenolysis, thus fostering hyperglycemia [37,38]. Indeed, rats on a chronic HFD exhibit significant metabolic disturbances, such as impaired glucose tolerance and elevated blood glucose levels.

Our findings in HFD rats affirm the significance of this model for investigating obesity, hyperglycemia, IR, and NAFLD. Notably, CA demonstrated a dose-dependent capacity to improve hepatic morphology and mitigate hepatic steatosis without impacting food intake, caloric consumption, fat, or body weight gain. Furthermore, CA did not lower fasting glucose or insulin levels nor enhance glucose tolerance, indicating that its protective effects are not associated with anti-adiposity or hypoglycemic properties. Rather, these hepatic benefits are likely hepatic-centric mechanisms that are mediated by several mechanisms that include hypolipidemic, antioxidant, and anti-inflammatory actions. Supporting this, previous research showed that CA did not stimulate glucose uptake in C2C12 muscle cells [39] or induce insulin release from isolated pancreatic β-cells at baseline glucose levels [40]. Conversely, CA at concentrations of 5 and 10 mg/kg improved glucose tolerance and reduced fasting glucose in nonobese rats with T2DM induced by high doses of STZ [41]. CA also enhanced insulin receptor phosphorylation and signaling in FL83B liver cells [42]. However, other studies have suggested that CA derivatives, such as ferulic acid and caffeic acid, exert anti-obesity and hypoglycemic effects by modulating key enzymes involved in adipose tissue lipogenesis and hepatic glucose synthesis, along with stimulating insulin release [24]. Moreover, our results contrast with those of Wang et al. [43] and Lee et al. [44], who reported that CA at 40 mg/kg effectively reduced body weight and adipose tissue expansion in HFD-fed mice, and with Mnafgui et al. [45], who observed a reduction in body weight, hyperlipidemia, and hepatic steatosis in HFD Wistar rats after 7 weeks of CA treatment at 30 mg/kg. Additionally, in vitro research by Kang et al. [46] highlighted CA’s potential to stimulate white fat browning in 3T3-L1 adipocytes. These discrepancies likely arise from variations in animal models (mice versus rats; HFD vs. STZ), dietary compositions of the HFD, treatment durations (12 weeks versus 7 weeks), sources of CA, and differences between in vivo and in vitro methodologies.

Our findings on body weight, food intake, and glucose and insulin intolerance in HFD rats also validate and affirm the significance of this model for obesity, hyperglycemia, IR, and NAFLD. On the other hand, the use of CA as a possible treatment for NALFD exhibited a dose-dependent ability to improve hepatic morphology and reduce hepatic steatosis without affecting food intake, caloric consumption, fat, or body weight gain. Additionally, CA did not lower fasting glucose or insulin levels, nor enhance glucose tolerance, indicating its protective effects are not linked to anti-adiposity or hypoglycemic properties or through modulating insulin action. Instead, these benefits seem likely to arise from hepatic-centric mechanisms, including hypolipidemic, antioxidant, and anti-inflammatory actions. Notably, previous studies demonstrated that CA did not stimulate glucose uptake in C2C12 muscle cells [39] or induce insulin release from isolated pancreatic β-cells [40]. In contrast to our data, CA at 5 and 10 mg/kg improved oral glucose tolerance tests and reduced fasting glucose in non-obese rats with T2DM induced by high-dose STZ [41] and enhanced insulin receptor signaling in FL83B liver cells [42]. Other studies suggest that CA derivatives, such as ferulic acid and caffeic acid, exhibit anti-obesity and hypoglycemic effects by modulating key enzymes in adipose tissue lipogenesis and hepatic glucose synthesis [24]. Additionally, our results contrast with Wang et al. [43], who reported that CA at 40 mg/kg effectively reduced body weight and adipose tissue in HFD-fed mice, and Mnafgui et al. [45], who noted reductions in body weight, hyperlipidemia, and hepatic steatosis in HFD Wistar rats after 7 weeks of CA treatment at 30 mg/kg. Furthermore, Kang et al. [46] highlighted CA’s potential to stimulate white fat browning in 3T3-L1 adipocytes. These discrepancies may stem from differences in animal models (mice versus rats), dietary compositions, treatment durations (12 weeks versus 7 weeks), sources of CA, and distinctions between in vivo and in vitro methodologies.

Yet, NAFLD remains a multifaceted condition driven by the interplay of hepatic oxidative stress, inflammation, hyperlipidemia, apoptosis, and peripheral IR. Peripheral IR, which is associated with obesity, leads to an impaired ability of insulin to regulate glucose and lipid metabolism, resulting in elevated levels of FFAs released from adipose tissue [47]. This influx of FFAs into the liver exacerbates lipid accumulation, contributing to hepatic lipotoxicity and steatosis [47,48,49]. Elevated FFAs are subject to β-oxidation, thus impairing mitochondria and generating massive amounts of ROS that overwhelm the liver’s antioxidant defenses and trigger oxidative stress [36]. The ROS and oxidative damage instigate an inflammatory response, with activated Kupffer cells and hepatic stellate cells releasing pro-inflammatory cytokines such as TNF-α and IL-6, further aggravating liver injury [36,49]. In addition, ROS is the key mediator in NALFD inflammation and apoptosis in NAFLD by upregulating the NF-κB, a master inflammatory transcription factor, and p53, a tumor suppressor gene that activates the p53/Bax apoptotic mitochondria pathway [48,50]. The ROS and inflammatory milieu not only perpetuate oxidative stress but also promote hyperlipidemia by enhancing lipogenesis through the upregulation of key proteins and enzymes like SREBP-1c and FAS [51,52,53,54]. Therefore, the relationship between peripheral IR and these hepatic processes forms a vicious cycle in which IR promotes increased FFAs, leading to steatosis and oxidative stress, which in turn fuels inflammation and apoptosis.

Our findings further elucidate the roles of oxidative stress, inflammation, and apoptosis in the pathogenesis of NAFLD resulting from an HFD. We observed a significant increase in lipid peroxides, coupled with reduced antioxidant levels, thereby confirming the presence of oxidative stress in the livers of HFD rats. Additionally, hepatocyte ballooning was noted, which correlated with dyslipidemia and heightened hepatic TGs and CHOL synthesis. Liver damage and apoptosis were evidenced by elevated serum levels of liver function enzymes, including AST, ALT, and γ-GT, as well as decreased Bcl-2 and increased Bax and caspase-3 levels. These results are consistent with other studies exploring the molecular mechanisms underpinning NAFLD, both in vivo and in vitro [55,56,57,58,59]. Conversely, CA effectively mitigated these pathological changes in the livers of HFD rats. It enhanced levels of GSH, SOD, HO-1, and Bcl2, while concurrently reducing hepatic TGs and CHOL levels in both control and HFD rats. These findings suggest that CA exhibits robust antioxidant and hypolipidemic properties under both basal and metabolic conditions, potentially offering a novel therapeutic strategy against oxidative and inflammatory disorders. Notably, while CA elevated antioxidant levels and reduced lipid synthesis and accumulation in the livers of both control and HFD rats, it selectively reduced the expression of NF-κB and levels of inflammatory cytokines, Bax, and caspase-3 exclusively in HFD rats. These results suggest that CA acts to suppress NASH by diverse, interconnected mechanisms, namely, stimulating antioxidants, suppressing lipogenesis and fat accumulation, and upregulating Bcl2, which accounts for its secondary inhibitory effects on inflammation and apoptosis.

Supporting evidence from various studies highlights CA’s remarkable capacity to diminish TG and CHOL synthesis and accumulation in oleic acid-treated HepG2 cells and in the livers of HFD-fed rats [29,43,45]. Furthermore, CA has been shown to mitigate acetic acid-induced inflammatory colitis by inhibiting the TLR4/NF-κB/TNFα and IL-6 pathways [60]. It also reduced adipose tissue inflammation by downregulating TNF-α expression in HFD-fed mice [44]. Similar inhibitory effects on NF-κB, IL-6, and TNF-α have been observed in Dextran Sodium Sulfate (DSS)-induced ulcerative colitis models [61]. Additionally, CA prevented depressive-like behavior and brain damage in lipopolysaccharide (LPS)-treated rats by upregulating GSH and SOD while concurrently suppressing MDA, IL-6, and TNF-α [62]. CA nanoparticles have also demonstrated the ability to reduce inflammation and apoptosis in acute hepatitis models by decreasing levels of TNF-α, IL-1β, and IL-18, as well as inhibiting NF-κB, NLRP3, and caspase-1, while promoting Bcl-2 levels [32]. Moreover, CA preserved GSH levels and other antioxidant enzymes, including catalase and glutathione peroxidase (GPx), while reducing IL-6 and MDA levels and inhibiting NF-κB activation in high-glucose-exposed HepG2 cells [63]. CA also prevented cisplatin-induced renal damage by decreasing MDA levels and increasing GSH, SOD, and GPx levels [26]. Nonetheless, derivatives of CA have been identified as potent scavengers of ROS [64]. Additionally, CA prevented liver and kidney damage in gentamicin-treated rats by suppressing inflammation and enhancing SOD, GPx, and catalase expression [27].

Our findings prompted a deeper exploration into the molecular mechanisms through which CA exerts its hypolipidemic and antioxidant effects, major effects observed in the livers of control and HFD rats. The transcription factor SREBP1c is vital for the synthesis of hepatic fatty acids and triglycerides, promoting key lipogenic enzymes such as ACC and FAS [65]. Conversely, PPARα facilitates mitochondrial fatty acid oxidation, counteracting SREBP1 activity [66]. In NAFLD, increased hepatic mRNA levels of SREBP1, FAS, and ACC were observed were accompanied by decreased PPARα expression [65,67,68]. Conversely, strategies that upregulate PPARα or inhibit SREBP1c activity have been shown to alleviate hepatic steatosis and liver damage in obese and diabetic models [53,54,65,66]. Similarly, enhancing antioxidant expression is a promising approach for preventing and treating NAFLD [69]. Consequently, we focused on Nrf2 due to its pivotal role in regulating oxidative stress, fatty acid oxidation, and lipogenesis. The primary function of nuclear Nrf2 activation is to elevate antioxidant expression to combat oxidative stress [70]. Nrf2 activators have been shown to inhibit hepatic lipogenesis by decreasing the SREBP1, FAS, and ACC1 [12,71,72,73]. Moreover, Nrf2 enhances FFAs and oxidation by regulating CD36 expression and upregulating PPARα and β-oxidation enzymes [13,19,74,75,76,77]. Additionally, Nrf2 can inhibit apoptosis by upregulating the anti-apoptotic protein Bcl-2 [78]. In line with the antioxidant effects of CA, we also found that CA’s hypolipidemic action was associated with the suppression of SREBP1, FAS, and ACC, alongside the stimulation of PPARα. This observation is corroborated by Wu et al. [29], who demonstrated that CA directly alleviates hepatic lipid synthesis and accumulation in oleic acid-treated HepG2 cells and HFD-fed rats by downregulating SREBP1, ACC, FAS, and SCD1 while enhancing the mRNA levels of PPARγ, PGC1α, and CPT1A. Wang et al. [43] similarly confirmed CA’s efficacy in lowering TG and CHOL levels in HFD rats and oleic acid-treated HepG2 cells. In the same manner, CA reduced serum TG, CHOL, and LDL-c levels in HFD-fed rats, which were correlated with decreased serum lipase activity [45].

Importantly, our novel findings suggest that the antioxidant, hypolipidemic, and anti-apoptotic effects of CA are Nrf2-dependent. Moreover, CA stimulated Nrf2 nuclear translocation without altering its mRNA levels in the livers of both control and HFD-fed rats. Treating HFD + CA rats with brusatol abolished CA’s stimulatory effects on SREBP1, FAS, ACC, as well as on GSH and other antioxidants, thereby negating all protective effects of CA in HFD models. On the other hand, FFAs, glucose, and inflammatory cytokines, which are potent inducers of SREBP1 expression [52,53,54,79], play a significant role under insulin resistance and metabolic disturbances. The regulatory influence of Nrf2 on SREBP1 expression and activation may clarify the association of these markers with CA’s hypolipidemic effect. Notably, CA treatment also suppressed SREBP1, FAS, and ACC in the livers of control rats, despite the presence of elevated FFAs, hyperglycemia, and inflammatory cytokines dissipating their role in the stimulatory effect of CA on proteins of lipid synthesis. Also, the increased Nrf2 activity in the livers of control rats likely accounts for the observed elevated levels of Bcl-2, GSH, SOD, and HO-1. However, given that ROS also stimulates SREBP1 and other fatty acid synthesis genes [51], it is plausible that Nrf2 mediates the repression of these lipogenic genes by reducing ROS levels in both control and HFD-fed rats.

Nrf2 internal activity depends mainly on the cytoplasmic stability and its ability to translocate and stay in the nucleus for a longer time. This regulation depends on cytoplasmic and nuclear proteins. In the cytoplasm, Keap1 is the most common negative regulator of Nrf2. Keap1 1 binds tightly to Nrf2 in the cytoplasm and stimulates Nrf2 proteasome degradation. Under IR conditions, hyperglycemia plays a significant role in inducing Nrf2 degradation in the liver by upregulating Keap1 or through activating other non-Keap1 degradation pathways such as glycogen synthase kinase 3 (GSK3), which induces phosphorylation-mediated degradation of Nrf2 [80]. In line with this evidence, the reduction in the nuclear activities of Nrf2 in the livers of HFD-fed rats was associated with a concomitant increment in the mRNA of Keap1, which could be due to obesity-induced IR and hyperglycemia. However, our study indicates that the stimulatory role of CA on Nrf2 nuclear transactivation is mediated by suppressing the expression of Keap1, thus reducing its association with Nrf2. Of course, this is glucose-independent, given that CA did not affect glucose levels but reduced the mRNA expression of Keap1 in the livers of the control and HFD rats. This is a unique finding and is supported by many other observations, which have shown the ability of multiple plant-derived phytochemicals to stimulate Nrf2 activation by modulating the expression of Keap1. Based on these data, we can conclude that CA stimulates Nrf2 nuclear translocation by suppressing Keap1 [66].

## 4. Materials and Methods

### 4.1. Animals

Wistar albino male rats (100 ± 15 g) were obtained from the animal facility at King Saud University (Riyadh, Saudi Arabia). Animals were housed in standard polypropylene cages (40 × 25 × 15 cm^3^) with three rats per cage, under controlled environmental conditions (temperature: 20–22 °C; humidity: 40–60%; 12 h light/dark cycle). Rats were provided with standard chow and water ad libitum throughout the study. Only male rats were used to avoid hormonal variability associated with the estrous cycle in females, which may influence metabolic, inflammatory, and oxidative stress responses, thereby ensuring experimental consistency. Following a 1-week acclimatization period, rats were randomly assigned to six experimental groups (*n* = 8 per group) using a computer-generated randomization schedule. To minimize bias, outcome assessments—including biochemical, molecular, and histological analyses—were performed by investigators blinded to group allocation. Blinding was maintained using coded sample labeling prepared by an independent individual not involved in the data collection or analysis. All experimental procedures were approved by the Research Ethics Committee at King Saud University, Riyadh, Saudi Arabia (IRB# KSU-SE-24-35, approval date: 6 June 2024).

### 4.2. Control and HFD Composition

To induce obesity in Wistar rats, we implemented a high-fat diet (HFD) from Research Diets Inc. (New Brunswick, NJ, USA, Catalog ID D12492), which comprised 35% fat and had a caloric density of 5.24%. This formulation included 245 g/kg of fat and 25 g/kg of soybean oil, significantly surpassing the standard diet (Catalog ID D12450K), which contained only 4.3% fat and a caloric density of 3.85 kcal/g. Additionally, the HFD featured 172.8 g/kg of sucrose, enhancing its caloric content, while the standard diet lacked sucrose. Both diets were formulated with essential ingredients, including casein, corn starch, maltodextrin, and vital vitamins and minerals, as detailed in Table 4. The rats were acclimatized for one week to alleviate stress before initiating the dietary regimen designed to model obesity. This HFD has been effectively utilized in our laboratory to induce obesity in this strain over a 12- to 16-week chronic feeding period [81,82].

### 4.3. Drugs

Natural CA powder (C_6_H_5_CH=CHCOOH) with a purity of ≥99% (W228826) and Carboxymethylcellulose (CMC) (Catalog #C5678) were procured from Sigma Aldrich, St. Louis, MO, USA. For each experimental trial, CA was freshly prepared by dissolving the powder in a 0.5% CMC solution to obtain the desired concentration.

### 4.4. Experimental Groups and Design

Previous studies demonstrated that treatment with CA at increasing dosages of 20 and 40 mg/kg attenuated brain damage in diabetic mice by alleviating oxidative stress and inflammation [83]. Additionally, the in vivo application of brusatol to inhibit the nuclear activation of Nrf2 across various rat tissues is well reported [84,85]. Based on this, the experimental framework of this study comprised six distinct groups of rats, each consisting of randomly selected eight animals. The first group served as the control group, maintained a standard diet, and administered a 0.5% carboxymethylcellulose (CMC) solution as the control vehicle. The second group received the standard diet supplemented with a maximum dosage of CA at 40 mg/kg. The third group was placed on an HFD while concurrently treated with the vehicle (0.5% CMC). In the fourth and fifth groups, rats were fed HFD alongside CA at either a dosage of 20 mg/kg or 40 mg/kg. The sixth group was provided with an HFD and co-treated with brusatol (2 mg/kg), a selective Nrf2 inhibitor, one hour before receiving CA (40 mg/kg). Both CA and brusatol were administered twice per week (every three days) throughout the study. CA was suspended in 0.5% carboxymethylcellulose (CMC) and administered orally by gavage at a volume of 1 mL per 100 g of body weight, while brusatol was administered intraperitoneally, consistently two hours prior to CA administration. This comprehensive treatment protocol was conducted over a span of 12 weeks.

### 4.5. Measurement of Obesity-Related Anthropometric Parameters

In the twelfth week of the study, various anthropometric parameters were evaluated to assess obesity in rats. These included height, total body weight, and body mass index (BMI), calculated using the formula [body weight (g)/length^2^ (cm^2^)], in line with established protocols from previous research [86].

### 4.6. Intraperitoneal Glucose Tolerance Test (IPGTT) and Intraperitoneal Insulin Tolerance Test (IPITT)

At the end of the intervention period, both IPGTT and IPITT were performed to assess metabolic functions across all rat groups. The IPGTT was executed on day 80, following a 12 h fasting period, after which glucose was administered intraperitoneally at a dosage of 1.5 g/kg body weight (G8270, Sigma Aldrich, Dorset, UK). Two days later, the IPITT was conducted, necessitating a 4 h fasting period before the intraperitoneal administration of insulin at 0.75 IU/kg body weight (I9278-5ML, Sigma Aldrich, UK). During blood sample collection for both tests, blood glucose levels were measured at 0, 30, 60, 90, and 120 min, with 300 µL samples taken from the tail vein. To facilitate blood flow, the tails were pre-warmed under a heat lamp set to 37 °C for 5 min. In cases where stress reduction was necessary, mild isoflurane anesthesia was utilized. All blood samples were collected into EDTA-coated polypropylene microcentrifuge tubes and subsequently centrifuged at 1200× *g* for 10 min to isolate plasma. Blood glucose and insulin concentrations in the plasma samples were measured using rat-specific colorimetric and ELISA kits as per the provided instructions (81693 and 62300, Crystal Chem, Elk Grove, IL, USA). The blood glucose and insulin levels recorded at 0 min during the IPGTT were considered fasting levels and were employed to calculate homeostasis model assessment (HOMA) levels using the following formula: [plasma glucose (mg/dL) × insulin (ng/mL)/405].

### 4.7. Euthanasia, Blood Sampling, and Tissue Collection Procedures

Three days post the tolerance tests, the rats underwent an overnight fasting period before being anesthetized with a combination of ketamine and xylazine at dosages of 80 mg/kg and 10 mg/kg, respectively. Blood samples were collected via cardiac puncture into plain tubes. Following a 30 min clotting interval at room temperature, the samples were centrifuged at 1200× *g* for 10 min to isolate serum, which was then stored at −20 °C for subsequent analysis. All euthanasia procedures adhered to stringent ethical guidelines, including the practice of neck dislocation. Additionally, liver tissue and various adipose depots—namely subcutaneous (inguinal), epididymal, peritoneal, and mesenteric fat pads—were meticulously excised, weighed, cut into smaller parts, and immediately frozen at −80 °C for any further use.

### 4.8. Preparation of Liver Homogenates

Preparation of liver tissue homogenate followed previous evidence [87]. Liver homogenates from frozen rat samples were prepared with careful precision to ensure accuracy and consistency in the processing of specimens. Initially, 40 mg of frozen liver tissue was weighed and kept on ice to prevent protein degradation. Homogenization was achieved by adding ice-cold phosphate-buffered saline (PBS) to tissue at a ratio of 1:10 (*w*/*v*) (i.e., 40 mg/400 µL PBS). This tissue/PBS mixture was further supplemented with a protease and phosphatase inhibitor cocktail (ab271306, Abcam, Cambridge, UK) to achieve a final volume of 500 µL, thereby preserving protein stability. The tissue was finely minced and introduced into a pre-chilled homogenizer containing the buffer. Homogenization was performed on ice using a high-speed homogenizer set to 10,000 rpm for 30 s, ensuring a thorough and uniform suspension. Subsequently, the mixture was centrifuged at 1200× *g* for 10–15 min at 4 °C to eliminate cellular debris. The supernatant obtained was aliquoted into pre-chilled microcentrifuge tubes and stored at −80 °C for subsequent biochemical analysis.

### 4.9. Isolation of Nuclear Proteins from Frozen Liver Tissue

For the isolation of nuclear proteins, the Active Motif Nuclear Extract Kit (40010, Active Motif, Tokyo, Japan) was used [87], which utilizes 50 mg of tissue. In brief, the liver sample was thawed and finely chopped to facilitate homogenization in 500 µL of ice-cold PBS, which was enhanced with 1 mM sodium orthovanadate and 1 mM sodium fluoride to maintain protein integrity. The resulting homogenate was subsequently mixed with 500 µL of a hypotonic lysis buffer composed of 10 mM HEPES (pH 7.9), 10 mM KCl, 1.5 mM MgCl_2_, and 0.5% NP-40. After a 15 min incubation on ice, centrifugation at 1000× *g* for 5 min at 4 °C was performed to separate the cytoplasmic and nuclear components. The supernatant, which contained the cytoplasmic proteins, was carefully collected and stored at −80 °C for future analysis. The nuclear pellet was resuspended in 100 µL of a detergent-free lysis buffer consisting of 20 mM HEPES (pH 7.9), 400 mM NaCl, and 1.5 mM MgCl_2_, along with a protease inhibitor cocktail. After a 30 min incubation on ice with periodic vortexing, the mixture was centrifuged at 14,000× *g* for 10 min at 4 °C, yielding a clear nuclear extract.

### 4.10. Comprehensive Biochemical Assessments of the Serum

Total cholesterol (CHOL) levels were quantified using the ECCH-100 kit from BioAssay Systems (Hayward, CA, USA), as well as low-density lipoprotein cholesterol (LDL-c) and Triglyceride (TG) (MBS702165, MBS726298, respectively). Other ELISA kits were used to measure levels of tumor necrosis factor-alpha (TNF-α) from ThermoFisher (BMS622, Dreieich, Germany) and interleukin-6 (IL-6) from R&D Systems (R6000B, Minneapolis, MN, USA). Additionally, serum enzymatic activity was assessed for alanine aminotransferase (ALT), gamma-glutamyl transpeptidase (GGT), and aspartate aminotransferase (AST) using MyBioSource kits (San Diego, CA, USA, ALT: MBS269614; GGT: MBS9343646) and Cosmo Bio (Tokyo, Japan, AST: CSB-E13023r-1). All analyses were performed in duplicate (*n* = 8 per group), adhering rigorously to the manufacturers’ protocols to ensure the accuracy and reliability of the results.

### 4.11. Comprehensive Biochemical Assessments of the Liver Homogenates

Various biomarkers in liver samples were quantified using ELISA kits. The levels of IL-6 were determined with a kit from Elabscience (TX, USA; E-EL-R0015), while those of TNF-α were assessed using ThermoFisher’s kit (UK; BMS622). Malondialdehyde (MDA) concentrations were measured using a kit from AFG Scientific (Northbrook, IL, USA; EK720188). The levels of GSH were assessed with a kit from the same manufacturer (EK720816). However, the levels of HO-1 were quantified using AFG Scientific’s kit (EK720658). The levels of SOD were determined with a kit from AFG Scientific as well (EK720889). Apoptosis-related proteins, including B-cell lymphoma 2 (Bcl-2), Bcl-2-associated X protein (Bax), and cleaved caspase-3 were quantified in hepatic homogenates via ELISA (#ER0762, #ER0512, and ##ER2018, FineTest, Wuhan, China). All assays were performed according to the manufacturers’ guidelines, with each measurement conducted in duplicate from a sample size of *n* = 8 rats per group to ensure the validity of the results.

### 4.12. Biochemical Evaluation of Nuclear Tissue Fractions

The nuclear levels of Nrf2 were evaluated using an Nrf2 rat-specific kit from Abcam (Cambridge, UK; catalog number: ab207222). The nuclear levels of NF-κB were measured using a rat-specific ELISA kit (Cat# MBS752046, MyBioSource). Each experimental group included 8 samples, and all procedures adhered rigorously to the protocols established by the respective manufacturers to ensure the accuracy and reliability of the results.

### 4.13. Quantitative PCR Protocol for Gene Expression Analysis in Hepatic Tissues

In this study, the mRNA expression of key genes (e.g., keap1, Nrf2, SREBP1, FAS, ACC, and PPARα) was evaluated in hepatic tissues through real-time PCR analysis. Frozen liver samples, approximately 50 mg each, were first homogenized in TRIZOL reagent for RNA extraction. After incubation and phase separation, RNA was precipitated with isopropanol, washed with 75% ethanol, and resuspended in RNase-free water. Subsequently, 1 µg of RNA was reverse transcribed into cDNA using the K1621 cDNA Synthesis Kit from Thermo Fisher, following the manufacturer’s instructions. For quantitative PCR, reactions were prepared using SsoFast EvaGreen Supermix (Bio-Rad, Hercules, CA, USA), which included specific primers for each target gene and cDNA template. The amplification was performed on a CFX96 Real-Time PCR System, with the following cycling conditions: an initial denaturation at 95 °C for 2 min, followed by 40 cycles of denaturation at 95 °C for 5 s, and annealing/extension at 60 °C for 30 s. Expression levels were normalized to the reference gene β-actin, and relative quantification was performed using the ΔΔCt method. Each gene was assessed in duplicate with six biological replicates per experimental group, ensuring robust and statistically significant results (Table 5).

### 4.14. Histological Study

Hematoxylin and eosin (H&E) staining is a widely used histological technique for visualizing tissue morphology, including that of liver samples. First, formalin-fixed liver tissues were dehydrated using a series of graded alcohols, followed by embedding in paraffin wax blocks. Sections of 5–7 μm thickness were cut using a microtome and mounted on glass slides. Deparaffinization of the sections was achieved by placing them in xylene and rehydrating them through descending grades of ethanol. Subsequently, the slides were immersed in hematoxylin solution for 5–10 min to stain nuclei. The slides were then placed in HCL-alcohol to remove excess stain, followed by a brief rinse in tap water. Next, an eosin solution was applied to stain the cytoplasm and extracellular matrix for 1–3 min. Dehydration was performed through ascending grades of alcohol, clearing in xylene, and mounting with a coverslip using a mounting medium. Slides were examined under a light microscope. The entire procedure ensures optimal staining quality and preservation of tissue architecture for histopathological analysis.

### 4.15. Statistical Analysis

All collected data underwent statistical analysis using GraphPad Prism (Version 8). Normality was assessed with the Kolmogorov–Smirnov test. Analysis was conducted using one-way ANOVA, and significance levels were determined using Tukey’s post hoc test (*p* < 0.05). Results are presented as means ± standard deviation (SD) for all data sets.

## 5. Conclusions

Our study highlights the therapeutic potential of CA in mitigating hepatic steatosis and inflammation associated with NAFLD. By reducing oxidative stress and inhibiting key lipogenic pathways through Nrf2 activation, CA presents a promising strategy for liver health interventions. Importantly, these findings suggest that CA may be utilized as a complementary treatment for NAFLD, focusing on its hypolipidemic and antioxidant properties rather than weight reduction or glucose metabolism. As a prospective target for future studies, it will be important to validate cinnamic acid’s hepatoprotective efficacy across diverse NAFLD paradigms, such as the methionine–choline-deficient diet model or genetically predisposed mouse strains, to ascertain its activity against differing etiologies, and to explore combination regimens pairing CA with established insulin sensitizers or complementary antioxidants in order to assess potential synergistic benefits on lipid metabolism, oxidative stress, and inflammatory signaling. In addition, future clinical applications could harness CA’s mechanisms to develop targeted therapies that address the underlying pathophysiology of NAFLD, potentially improving patient outcomes and quality of life.

### Study Limitations

Although this study provides novel insights into the hepatoprotective effects of cinnamic acid (CA), it has several limitations that should be taken into consideration. First, the high-fat diet (HFD) mouse model may not fully mimic the genetic, environmental, and lifestyle complexities that contribute to non-alcoholic fatty liver disease (NAFLD) in humans. Furthermore, the 12-week treatment period in this study, while proven sufficient to induce measurable biochemical and histological changes, may not be enough to assess the long-term efficacy, safety, and adverse effects of cinnamic acid. Second, although this study demonstrated Nrf2 activation via nuclear extract assays and transcriptional changes, we did not perform protein-level validation (e.g., Western blot or immunohistochemistry) to confirm Nrf2 expression patterns and its subcellular location. This, in turn, limits the mechanistic depth of our conclusions regarding this pathway. Third, our histological analysis relied on qualitative descriptions of steatosis and cavitation without standardized quantitative assessments such as morphometric quantification of vacuolar space or progressive steatosis scoring, which would have provided a more objective measure of tissue damage. Fourth, methodological variability among published studies, including differences in dietary composition, animal species used, dosing regimen, and treatment duration, complicates direct comparisons and may affect the broader applicability of our findings. Fifth, the study was limited to male mice to minimize hormonal variation. Consequently, the generalizability of CA’s effects across genders remains to be determined. Future studies will include male and female cohorts to determine any gender-specific responses. Sixth, while our sample size (*n* = 8 per group) is consistent with common clinical practice, we did not perform a priori power or effect size calculations, which may limit the interpretation of our results. Similarly, although the Kolmogorov–Smirnov test was used to assess the normality of the data, more precise approaches—such as the Shapiro–Wilk test—may be preferable for smaller sample sizes. Additionally, another potential limitation of the current study is the absence of a high-fat control group, which would have helped determine whether the observed hepatic effects of CA were due solely to its direct pharmacological effects or were partially influenced by potential reductions in caloric intake. However, in our study, food and calorie intake did not differ significantly between groups, and the protective effects were dose-dependent and abolished by Nrf2 inhibition. Therefore, we interpreted these effects of cinnamic acid as primarily due to direct activation of the Nrf2 pathway. Finally, we did not determine the pharmacokinetic properties or systemic bioavailability of CA. In the absence of data on absorption, metabolism, and excretion, it remains unclear whether the dosing regimen used here can achieve therapeutically relevant concentrations in vivo. Therefore, filling these gaps in future studies will be important to enhance the translational potential of CA in the management of NAFLD.

## Figures and Tables

**Table 1:** Analysis of some obesity markers in all groups of rats.

Parameter	Control	Control + CA (40 mg/kg)	HFD	HFD + CA (20 mg/kg)	HFD + CA (40 mg/kg)	HFD + CA (40 mg/kg) + Brusatol
Initial body weights (g)	121.4 ± 9.4	120.1 ± 9.9	116.4 ± 7.8	118.3 ± 7.4	129.3 ± 6.4	110.2 ± 10.4
Final body weights (g)	438.3 ± 41.4	441.3 ± 39.7	584.5 ± 61.2 ^a^	568.4 ± 48.8 ^ab^	565.4 ± 54.3 ^ab^	571.3 ± 55.9 ^ab^
Weight gain (%)	292.3 ± 24.3	300.3 ± 29.5	447.8± 43.8 ^a^	425.5 ± 48.5 ^ab^	404.6 ± 38.6 ^ab^	469.2 ± 51.3 ^ab^
Cumulative food intake (last weeks/rat)	218.8 ± 21.7	224.5 ± 20.9	343.4 ± 28.6 ^ab^	356.6 ± 36.1 ^ab^	348.2 ± 36.7 ^ab^	348.8 ± 4.5 ^ab^
Calorie intake (kcal/last 2 weeks/rat)	842.7 ± 83.6	864.3 ± 80.4	1801.3 ± 149.0 ^ab^	1867.7± 189.7 ^ab^	1825.5 ± 192.3 ^ab^	1827.71 ± 23.58 ^ab^
BMI (g/cm^2^)	0.68 ± 0.05	0.65 ± 0.07	0.84 ± 0.08 ^ab^	0.86 ± 0.08 ^ab^	0.83 ± 0.09 ^ab^	0.85 ± 0.08 ^ab^
Mesenteric fat (g)	5.3 ± 0.56	5.9 ± 0.72	10.4 ± 1.1 ^ab^	11.3 ± 1.7 ^ab^	10.9 ± 1.1 ^ab^	11.2 ± 1.2 ^ab^
Subcutaneous fat (g)	7.8 ± 0.65	7.1 ± 0.73	12.5 ± 1.4 ^ab^	12.9± 1.4 ^ab^	11.8 ± 1.4 ^ab^	12.1 ± 1.6 ^ab^
Peritoneal fat (g)	4.6 ± 0.63	4.9 ± 0.59	8.6 ± 0.77 ^ab^	8.8 ± 0.85 ^ab^	8.4 ± 0.66 ^ab^	8.3 ± 0.99 ^ab^
Epididymal fat (g)	5.8 ± 0.55	5.6 ± 0.51	10.5 ± 1.3 ^ab^	11.5 ± 1.2 ^ab^	10.9 ± 0.98 ^ab^	11.2 ± 1.4 ^ab^
Total fat weight (g)	23.7 ± 2.4	23.1 ± 2.4	42.8 ± 5.04 ^ab^	44.9 ± 4.9 ^ab^	43.7 ± 5.8 ^ab^	42.6 ± 5.8 ^ab^
Adiposity index (%)	5.4 ± 0.71	5.7 ± 0.42	7.2 ± 0.81 ^ab^	7.8 ± 0.77 ^ab^	7.4 ± 0.84 ^ab^	7.5 ± 0.76 ^ab^

Normality was tested using the Shapiro–Wilk test. Data were analyzed by one-way followed by Tukey’s *t*-test as post hoc. Data are given as the means ± SD of 8 rats/group. The level of significance was shown as *p* < 0.05. a: significantly different vs. control; b: significantly different vs. control + CA (20 mg/kg).

**Table 2:** Comparative analysis of obesity-related metrics in all experimental groups of rats.

Parameter	Control	Control + CA (40 mg/kg)	HFD	HFD + CA (20 mg/kg)	HFD + CA (40 mg/kg)	HFD + CA (40 mg/kg + Brusatol
Fasting glucose (mg/dL)	96.9 ± 8.6	98.6 ± 6.5	202.2 ± 20.4 ^ab^	198.4 ± 18.4 ^ab^	206.3 ± 19.5 ^ab^	194.3 ± 19.3 ^ab^
Fasting Insulin (ng/mL)	3.9 ± 0.44	3.5 ± 0.53	7.6 ± 0.64 ^ab^	7.9 ± 0.66 ^abc^	7.4 ± 0.83 ^ab^	7.5 ± 0.72 ^ab^
HOMA-IR	0.91 ± 0.08	0.84 ± 0.07 ^a^	3.69 ± 0.44 ^ab^	3.86 ± 0.45 ^abc^	3.57 ± 0.33 ^bcd^	3.63 ± 0.63 ^abde^
Serum HbA1C (%)	4.32 ± 0.39	4.15 ± 0.43	7.41 ± 0.81 ^ab^	6.1 ± 0.77 ^abc^	4.6 ± 0.58 ^bcd^	7.9 ± 0.88 ^abde^
Serum Triglycerides (mg/dL)	83.3 ± 7.4	61.2 ± 5.8 ^a^	187.3 ± 16.5 ^ab^	123.4 ± 13.6 ^abc^	87.5 ± 8.6 ^bcd^	194.3 ± 18.5 ^abde^
Serum Cholesterol (mg/kg)	118.3 ± 11.4	85.6 ± 7.6 ^a^	256.4 ± 24.5 ^ab^	197.4 ± 17.6 ^abc^	124.5 ± 5.4 ^bcd^	248.5 ± 21.4 ^abde^
Serum LDL-c (mg/dL)	56.4 ± 6.2	41.2 ± 4.3 ^a^	125.4 ± 14.3 ^ab^	88.6 ± 9.4 ^abc^	59.6 ± 5.3 ^bcd^	119.4 ± 12.5 ^abde^
Hepatic Triglycerides (mg/g)	5.6 ± 0.45	4.1 ± 0.38 ^a^	13.8 ± 1.3 ^ab^	9.4 ± 0.84 ^abc^	6.1 ± 0.73 ^bcd^	12.8 ± 1.4 ^abde^
Hepatic Cholesterol (mg/g)	7.4 ± 0.68	5.8 ± 0.45 ^a^	17.5 ± 1.4 ^ab^	12.1 ± 1.2 ^abc^	7.5 ± 0.92 ^bcd^	20.4 ± 1.8 ^abde^

Normality was tested using the Shapiro–Wilk test. Data were analyzed by one-way followed by Tukey’s *t*-test as post hoc. Data are given as the means ± SD of 8 rats/group. Data are given as the means ± SD of 8 rats/group. The level of significance was shown as *p* < 0.05. a: significantly different vs. control, b: significantly different vs. control + CA (20 mg/kg), c: significantly different vs. HFD, d: significantly different vs. HFD+ CA (20 mg/kg), and e: significantly different vs. HFD+ CA (40 mg/kg).

**Table 3:** Analysis of Liver functions and Apoptotic markers in all groups of rats.

Parameter	Control	Control + CA (40 mg/kg)	HFD	HFD + CA (20 mg/kg)	HFD + CA (40 mg/kg)	HFD + CA (40 mg/kg) + Brusatol
Serum						
ALT (U/L)	31.6 ± 2.7	33.4 ± 3.1	92.4 ± 8.6 ^ab^	61.9 ± 6.3 ^abc^	35.6 ± 4.5 ^cd^	89.7 ± 9.8 ^abde^
AST (U/L)	44.5 ± 5.7	41.9 ± 5.5	112.3 ± 12.7 ^ab^	84.5 ± 8.8 ^abc^	46.8 ± 4.8 ^cd^	109.4 ± 10.8 ^abde^
γ-GT (U/L)	22.4 ± 1.7	19.8 ± 1.5	68.9 ± 5.7 ^ab^	41.7 ± 4.6 ^abc^	21.7 ± 1.6 ^cd^	64.5 ± 5.8 ^abde^
Liver						
BCl2 (pg/g protein)	54.1 ± 4.8	76.8 ± 6.6 ^a^	24.5 ± 2.1 ^ab^	36.6 ± 4.1 ^abc^	49.5 ± 3.7 ^bcd^	20.5 ± 1.3 ^abde^
Bax (pg/g protein)	21.9 ± 1.4	24.3 ± 2.7	108.5 ± 12.3 ^ab^	78.5 ± 8.8 ^abc^	32.4 ± 3.5 ^abcd^	113.5 ± 13.2 ^abde^
Caspase-3 (pg/g protein)	5.3 ± 0.83	4.9 ± 0.57	22.5 ± 1.8 ^ab^	12.4 ± 1.2 ^abc^	6.3 ± 0.74 ^bcd^	19.6 ± 1.9 ^abde^

Normality was tested using the Shapiro–Wilk test. Data were analyzed by one-way followed by Tukey’s *t*-test as post hoc. Data are given as the means ± SD of 8 rats/group. Data are given as the means ± SD of 8 rats/group. The level of significance was shown as *p* < 0.05. a: significantly different vs. control, b: significantly different vs. control + CA (20 mg/kg), c: significantly different vs. HFD, d: significantly different vs. HFD+ CA (20 mg/kg), and e: significantly different vs. HFD+ CA (40 mg/kg).

**Table 4:** Composition of both the control diet and high-fat diet (HFD) that is used in this study.

Product	Control Diet		HFD	
	gm%	kcal%	gm%	kcal%
Protein	19.2	20	26%	20%
Carbohydrate	67.3	70	26%	20%
Fat	4.3	10	35%	60%
Total		100		100
kcal/gm	3.85		5.24	
Ingredient composition of diet				
	gm	kcal	gm	kcal
Proteins				
Casein	200	800	200	800
L-Cystine	3	12	3	12
Carbohydrates				
Corn Starch	550	2200	0	0
Maltodextrin 10	150	600	125	500
Sucrose	0	0	68.8	500
Fibers				
Cellulose	50	0	50	50
Fats				
Soybean Oil	25	225	25	225
Fat	20	180	245	2205
Others				
Mineral Mix, S10026	10	0	10	0
DiCalcium Phosphate	13	0	13	0
Calcium Carbonate	5.5	0	5.5	0
Potassium Citrate, 1 H_2_O	16.5	0	16.5	0
Vitamin Mix, V10001	10	40	10	40
Vitamin Mix V10001C	0	0	0	0
Choline Bitartrate	2	0	2	0
				
FD&C Red Dye #40	0.025	0	0.05	0
FD&C Blue Dye #1	0.025	0	0	0

Total	1055.05	4057	773.85	4057

**Table 5:** Properties of the primers that are used in the real-time polymerase chain reaction.

Gene	Accession Number	Forward Primer (5′→3′)	Reverse Primer (5′→3′)	PB
*SREBP1c*	NM_001276707.1	CGGGACAGCTTAGCCTCTACA	CGGCCACAAGAAGTAGATCA	21
*Keap1*	NM_057152.2	CTTCGGGGAGGAGGAGTTCT	CGTTCAGATCATCGCGGCTG	132
*Nrf2*	NM_031789	AAAATCATTAACCTCCCTGTTGAT	CGGCGACTTTATTCTTACCTCTC3	118
*ACC1*	NM_022193.1	GCTGGGACAAAGAACCATCC	TCCGTTGTTGTGCATTATCTGG	193
*FAS*	NM_017332.1	CCACAGGACAAGCCCATCTT	TCGGAGACAGTTCACCAAGC	159
*NF-κB*	XM_342346.4	GTGCAGAAAGAAGACATTGAGGTG	AGGCTAGGGTCAGCGTATGG	176
*PPARα*	NM_0011453661	AAGTTTGAGTTTGCTGTGAAGTTCA	CGATGGGCTTCACGTTCAG	121
*β-actin*	NM_031144.3	AGGCCCCTCTGAACCCTAAG	CAGCCTGGATGGCTACGTACA	96

## Data Availability

The datasets used and analyzed during the current study are available from the corresponding author upon reasonable request.
